# From gene banks to farmer’s fields: using genomic selection to identify donors for a breeding program in rice to close the yield gap on smallholder farms

**DOI:** 10.1007/s00122-021-03909-9

**Published:** 2021-07-15

**Authors:** Ryokei Tanaka, James Lui-King, Sarah Tojo Mandaharisoa, Mbolatantely Rakotondramanana, Harisoa Nicole Ranaivo, Juan Pariasca-Tanaka, Hiromi Kajiya Kanegae, Hiroyoshi Iwata, Matthias Wissuwa

**Affiliations:** 1 Department of Agricultural and Environmental Biology, Graduate School of Agricultural and Life Sciences, The University of Tokyo, 1-1-1 Yayoi, Bunkyo, Tokyo 113-8657 Japan; 2 International Program in Agricultural Development Studies, Graduate School of Agricultural and Life Sciences, The University of Tokyo, 1-1-1 Yayoi, Bunkyo, Tokyo 113-8657 Japan; 3 Crop, Livestock and Environment Division, Japan International Research Center for Agricultural Sciences (JIRCAS), 1-1 Ohwashi, Tsukuba, Ibaraki 305-8686 Japan; 4 Rice Research Department, The National Center for Applied Research On Rural Development (FOFIFA), Antananarivo, 101 Madagascar

## Abstract

**Key message:**

**Despite phenotyping the training set under unfavorable conditions on smallholder farms in Madagascar, we were able to successfully apply genomic prediction to select donors among gene bank accessions**.

**Abstract:**

Poor soil fertility and low fertilizer application rates are main reasons for the large yield gap observed for rice produced in sub-Saharan Africa. Traditional varieties that are preserved in gene banks were shown to possess traits and alleles that would improve the performance of modern variety under such low-input conditions. How to accelerate the utilization of gene bank resources in crop improvement is an unresolved question and here our objective was to test whether genomic prediction could aid in the selection of promising donors. A subset of the 3,024 sequenced accessions from the IRRI rice gene bank was phenotyped for yield and agronomic traits for two years in unfertilized farmers’ fields in Madagascar, and based on these data, a genomic prediction model was developed. This model was applied to predict the performance of the entire set of 3024 accessions, and the top predicted performers were sent to Madagascar for confirmatory trials. The prediction accuracies ranged from 0.10 to 0.30 for grain yield, from 0.25 to 0.63 for straw biomass, to 0.71 for heading date. Two accessions have subsequently been utilized as donors in rice breeding programs in Madagascar. Despite having conducted phenotypic evaluations under challenging conditions on smallholder farms, our results are encouraging as the prediction accuracy realized in on-farm experiments was in the range of accuracies achieved in on-station studies. Thus, we could provide clear empirical evidence on the value of genomic selection in identifying suitable genetic resources for crop improvement, if genotypic data are available.

**Supplementary Information:**

The online version contains supplementary material available at 10.1007/s00122-021-03909-9.

## Introduction

The demand for rice in sub-Saharan Africa (SSA) is increasing steadily, outpacing local supply, and forcing many Africa countries to import increasing amounts of rice from Asia (USDA 2018). This growing shortage in local supply is due to the much lower average yields (2.3 t ha^−1^) achieved across Africa compared to Asia (4.8 t ha^−1^) (FAOSTAT 2018). The low grain yields in rice in SSA are caused by a combination of low fertilizer application rates with generally low soil fertility of the highly weathered soils which are typical throughout the region (Saito et al. 2019). Soils like the commonly encountered Oxisols are known to bind phosphorous (P) in forms that are not plant available, causing P to be the most frequent limiting nutrient in rice production in SSA (Saito et al. 2019). VanDamme et al. (2015) highlighted that a cost-efficient partial solution to the soil fertility problem in SSA would be the development of varieties with improved P acquisition and utilization efficiencies.

The abovementioned general trends for rice production and consumption also apply to Madagascar, the second biggest rice producer in SSA. Fertilizer applications have remained very low (Tsujimoto et al. 2019) despite standard recommendations for NPK fertilizers being in existence for decades. As a result, the national average yield remains below 3 t ha^−1^, whereas achievable on-farm yields can exceed 7 t ha^−1^ (Saito et al. 2017) and are therefore comparable to tropical regions elsewhere. This large yield gap highlights that conventional on-station breeding approaches that seek to select breeding lines with high yield potential under “ideal” high-input conditions may not produce desired results. The prevalence of traditional rice varieties throughout Madagascar (Minten and Barrett 2008) is a further sign that plant breeding has not properly addressed the needs of the mostly resource-poor smallholder farmers. It is furthermore indicative of specific adaptations to lower soil fertility being present in such traditional varieties, which were found to be more efficient in P acquisition (Mori et al. 2016) and internal P utilization (Wissuwa et al. 2015) or may even show a combination of both desirable traits (Rose et al. 2015). A future breeding program targeting to close this yield gap may move the selection process from highly fertilized breeding stations to fields representing conditions a crop may experience in farmers’ fields and should attempt to utilize any adaptive traits of traditional varieties.

That traditional varieties may contain useful genes and alleles for certain traits and may therefore serve as donors to improve such traits in modern breeding populations which have been a chief reason to collect and preserve such varieties in crop gene banks. The largest collection of rice genetic resources with more than 130,000 accessions is stored in the gene bank of the International Rice Research Institute (IRRI). One potential problem of exploiting this resource is its sheer size. Phenotyping thousands of lines will require resources in terms of land and labor that few projects and institutes can manage. It is therefore of importance for the utility of such resources to have enough information associated with accessions to allow for targeted selections of smaller sub-sets of accessions for more detailed phenotypic evaluations. One invaluable step in this regard was the establishment of the publicly available SNP-Seek database (https://snp-seek.irri.org) providing sequence variants and passport data of more than 3000 rice accessions (3 K accessions) of diverse origin and genetic background (Mansueto et al. 2017).

Such genotyping efforts allow for the implementation of genomic selection (Meuwissen et al. 2001) as a tool to identify promising accession from gene banks. Based on phenotypic values available for a subset of the 3 K accessions, their genotypic values can be predicted applying a statistical model that establishes relations between SNP genotype and phenotype. Using this genomic selection model to predict genotypic values of the untested accessions would allow for the identification of potentially promising donor accessions among the entire 3 K set without the need for testing all accessions in the field. This concept was first proposed by Pace et al. (2015) and demonstrated in a large-scale experiment using more than 1,000 sorghum accessions (Yu et al. 2016) and is becoming one of the important applications of genomic selection (Crossa et al. 2017). In a typical genomic selection, genotypes are selected based on having higher predicted genotypic values (PGVs). Tanaka and Iwata (2018) recently proposed an alternative selection criterion termed "expected improvement" (EI). Using simulations based on a diversity panel of rice, they demonstrated that the proposed EI-based selection can identify superior accessions more efficiently compared to the standard PGV-based selection.

Genomic selection has been evaluated in rice (e.g*.*, Onogi et al. 2015; Spindel et al. 2015) and in other crops such as wheat (e.g*.*, Crossa et al. 2010; Heffner et al. 2011) or maize (e.g*.*, Zhao et al. 2012; Cui et al. 2020). Typically, phenotypic evaluations were conducted in well-managed breeding stations under high-input conditions where efforts had been made to maximize heritability by minimizing the environmental variation, as was the case in the proof-of-concept study of Yu et al. (2016). To what extent genomic prediction would be capable of reliably identifying superior accessions under less uniform conditions and in the presence of abiotic stresses, as one may encounter in farmers’ fields, has not been investigated.

The logistics of conducting experiments in smallholder farmer fields can be challenging and typically place a size limit on experiments as a result of factors such a field size, presence of gradients, absence of machinery for precise land leveling, distance from laboratories or even electricity. While the entire 3 K set has been phenotyped on the IRRI research farm, such a task would be daunting on a smallholder rice farm in Madagascar. The potential of using a small subset from the 3 K accessions to build a genomic selection model in order to predict the performance of the entire 3 K set would therefore be a game-changer as the genotypic variation present in much larger sets of gene bank accessions would become accessible to applied breeding programs. The present study tests to what extent this approach would be feasible under the low-input conditions in smallholder rice farms in Madagascar where typically no fertilizers other than organic manures or composts are applied. Specifically, our objectives were: (1) to develop a GS model based on field data obtained in farmer’s fields in Madagascar, (2) to use the model to predict best accessions among the 3 K set and to import them from IRRI for confirmatory tests in the field, (3) compare the selection based on the "expected improvement" (EI) criterion to the standard PGV-based selection, and (4) to investigate whether a second cycle of improving the model and prediction accession performance increases the precision of this approach.

## Materials and methods

### Field experiments and selection methods

Field experiments were conducted in the central highlands of Madagascar over a 3-year period from 2016 to 2019. Experimental field sites used were at elevations between 950 and 1350 m, and experiments were conducted during the rainy season with sowing in November, transplanting in late November–December and harvests in April–May. A common characteristic of all experimental sites was that they were located in farmers’ fields, that no fertilizer was applied, which is the predominant farmer’s practice in the region, and that no supplementary irrigation was provided beyond what was available from rainfall and rainfed small creeks. Briefly, the flow of experiments was as follows: In the 2016–17 season (year 1), a diversity panel (set 1, *n* = 359) was selected from the set of 3,024 sequenced rice accessions (3 K panel; https://snp-seek.irri.org) publicly available at IRRI and phenotyped at two sites (Anjiro and Behenjy). Based on the phenotypic data, a genomic prediction model was developed and utilized to predict the performance of all 3 K panel accessions. Accessions predicted to perform well were selected together with a random control group, and both (*n* = 234) were imported from the IRRI gene bank and grown together with the original panel at two sites (Anjiro and Ankazo) in the 2017–18 season (year 2). Phenotypic data from these trials were used to update the prediction model, and best-performing accessions not already used previously were selected (*n* = 52) and imported from IRRI for year 3 confirmatory trials in the 2018–19 season.

Genotypes used were selected from the 3 K panel that is publicly available at IRRI. We targeted accessions of the *indica* subpopulation and first selected based on geographical origin. All available accessions from the following countries were selected: Madagascar (57), Sri Lanka (44), Nepal (37), Bhutan (18). The reason why we focused on the latter two countries was to include accessions with possible adaptation to highland environments, while Sri Lankan accessions may contribute some adaptation to problems of soils (e.g., iron toxicity). The remaining 203 accessions were selected to obtain a representative sample of the *indica* gene pool, avoiding closely related accessions using phylogenetic analyses tools provided in SNP-Seek website. From the total 359 accessions imported from IRRI into Madagascar in year 1, 22 belonged to the *japonica* subspecies (from Madagascar and Bhutan), 15 or 12 accessions belonged to the *aus* and *aromatic* subpopulations (from Nepal, Madagascar, Bhutan, respectively), and 2 were classified as *admix*. The remaining 298 accessions belonged to the *indica* group, and in total 29 countries were represented. Hereinafter, experimental details will be provided year by year and a diagram depicting the flow of materials used in evaluations and predictions in different years is given in supplementary Figure S1.

Experiments in year 1 were conducted with 359 accessions at two locations, Anjiro (elevation 950 m, 18°54′01.7″S 47°58′12.4″E) and Behenjy (elevation 1350 m, 19°10′48.3″S 47°29′46.3″E). In addition to low soil fertility and especially P deficiency, growth at the Behenjy site was limited by low temperatures and mild iron toxicity. At both sites, accessions were transplanted in 2-row micro-plots of 2 m length with spacing of 20 cm between and within rows (22 single plant hills per plot). Heading date (HD) was recorded at 50% heading rate for each accession. During harvest, five representative plants per plot were cut, and tiller number and plant height (in cm from the base to the tip of the flag leaf) were recorded. Panicles were separated from straw and straw weight (STW) determined. Panicles were taken to the laboratory and air-dried for a week before total panicle dry weight (TPW) was determined. Straw and panicle weights are given in grams per plant for all experiments.

Experiments in year 2 were conducted in Anjiro, and instead of the cold-affected Behenjy, the warmer site Ankazo at elevation 1150 m (19°40′07.9″S 46°33′53.9″E) was chosen. Plot size was as in year 1, but each site had a second replication in a randomized complete block design. Procedures were similar as in year 1, with the exception that straw of one of the 5 representative plants sampled was taken to the laboratory to determine straw dry weight. The measured moisture content in the straw of this plant was then used to estimate STW of the entire 5-plant sample. The new set of 234 accessions was selected using the predicted genotypic values (PGV) and expected improvement (EI) values from the GBLUP genomic prediction model (Eqs. 5–7) built on the phenotypic record obtained in the year 1. Out of the 234 accessions, 79 were selected for screening of superior accessions for STW or TPW. For this screening, the top 20 accessions were independently listed for each trait (STW and TPW) by using each criterium (PGV and EI) for each environment (Anjiro and Behenjy) and then merged into one list. Details of the selection result, including the overlap between the two criteria, are described in the Result section. Of the remaining 155 new accessions, 68 were chosen randomly as a control group and the remaining 87 were included to enhance the training data for genomic selection, considering the predicted STW and HD (supplementary Figure S2). In short, accessions were selected for early/middle/late predicted HD, and high/middle/low predicted STW. Furthermore, 289 out of the initial 359 accessions were evaluated again in the second year (the remaining 70 accessions were removed due to low adaptation in Madagascar in the year 1 experiment). In total, 523 accessions were evaluated in the field in year 2.

The experiment in year 3 was conducted on a smaller number of accessions, mainly aiming at screening accessions predicted to be superior for TPW. A genomic prediction model for TPW was built on the observed phenotypic values from Anjiro (year 1) and Ankazo (year 2) (Eq. 3). The observations from year 2 of Anjiro were not used for the selection because that field was flooded after a cyclone, and thereafter, the plants were no longer under P-limited condition. Using PGV or EI from the model, 42 accessions were selected by PGV and 45 accessions by EI from the untested subset of the 3 K panel (i.e., non-tested in our previous two years of experiment). Due to an overlap of 35 accessions between the 42 and 45 selected sets, a total of 52 accessions were selected in year 3. In addition to those 52 newly selected accessions, 23 accessions were evaluated as a control group. These 23 were chosen from accessions evaluated in both two previous years. In order to ensure a wide phenotypic (and genetic) variation, these check accessions were selected at equally spaced intervals (every 22nd–23rd accession) from the distribution of estimated genotypic values for TPW (i.e., fitted genotypic values calculated in Eq. 3). Predicted HD was taken into account to avoid selecting very late maturing accessions (not suitable during very dry or cool years). Thus, in total 75 accessions were evaluated in year 3 in Anjiro and Ankazo.

For replicated experiments in year 2 and 3, broad-sense heritability of each trait was estimated based on the model:

1 $$y_{ij} = g_{i} + r_{j} + e_{ij}$$

where ${y}_{ij}$ is the phenotypic value of the *i*th genotype in the *j*th replication, ${g}_{i}$ is the genotypic value of the *i*th genotype (modeled as a random effect), ${r}_{j}$ is the effect of the *j*th replication (modeled as a fixed effect), and ${e}_{ij}$ is the residual.

Heritability was estimated using

2 $$H^{2} = \frac{{V_{g} }}{{V_{g} + \frac{{V_{e} }}{R}}}$$

where ${V}_{g}$ is the estimated genotypic variance, ${V}_{e}$ the estimated residual variance, and $R$ the number of replications (therefore $R=2$ in our case).

### Genomic prediction

For each trait, the GBLUP model (VanRaden 2008) was used for predicting or estimating the genotypic values of untested or tested accessions:

3 $$y = X\beta + Zu + e, u \sim MVN\left( {0, G\sigma_{u}^{2} } \right), e \sim MVN\left( {0, I\sigma_{e}^{2} } \right)$$

where $y$ is a vector of phenotypic values, $X$ is a design matrix for the fixed effect, $\beta$ is a vector of fixed effects, $Z$ is a design matrix for the random effect, $u$ is a vector of genotypic values, $e$ is a vector of residuals, $G$ is a genomic relationship matrix, ${\sigma }_{\mathrm{u}}^{2}$ is a genotypic variance, and ${\sigma }_{\mathrm{e}}^{2}$ is a residual variance. A zero vector and an identity matrix were denoted as $0$ and $I$, respectively. Note that the fixed effect-related terms varied in cases. When applied to the year 1 phenotype data (remember that genomic prediction was separately performed for each location) to select accessions for the year 2 experiment, only an intercept $\mu$ was modeled as a fixed effect in the equation. Therefore, the following equation was used:

4 $$y = 1\mu + Zu + e$$

where $1$ represents vector of ones. On the other hand, when applied to the year 1 Anjiro and year 2 Ankazo phenotypes to select accessions for the year 3 experiment, we had two fixed effects, and therefore, Eq. (3) with $${\beta }^{T}=\left[\begin{array}{cc}{\beta }_{1}& {\beta }_{2}\end{array}\right]$$ was used, where ${\beta }_{1}$ is the intercept for year 1 Anjiro and ${\beta }_{2}$ is the intercept for year 2 in Ankazo. The predictions of the genotypic values ($u$) were then used for the PGV-based genomic selection.

When there were more than two environments, the observed phenotypic values were scaled with mean zero and variance one for each environment, before applying the model. This is because the raw observations were distributed in largely different ranges even for the same trait, and therefore, single variance component should not be assigned. This procedure might imply that the two location has similar heritability, which is unlikely but acceptable assumption for keeping our model simple.

A genomic relationship matrix (*G* matrix) was calculated by using the A.mat function implemented in rrBLUP package, without using the shrinkage option (Endelman 2011). The 404 K core SNPs dataset was downloaded from the IRRI SNP-Seek website (https://snp-seek.irri.org/_download.zul). SNP having more than 5% missing data or a minor allele frequency below 2.5% was removed, and finally 186,229 SNPs for 3,024 accessions were extracted. The remaining missing states were imputed by using Beagle v.4.1 (21Jan17.6 cc; Browning and Browning 2016).

Prediction accuracy was evaluated by tenfold cross-validation (*i.e.*, validation based on 90% of randomly chosen accessions as training data and 10% of remaining accessions as test data) within each year and location, and results are shown as the Pearson’s correlation coefficient between predicted and observed values. Each tenfold validation was repeated 10 times, and the average prediction accuracy and its standard deviation were estimated. Note that this validation was not performed for year 3 phenotype data because the number of accessions was too small.

In addition to the cross-validation, the prediction accuracy for year 1 and year 2 experiments was also evaluated. For this validation, year 1 phenotype data were used to predict year 2 phenotypes. Accuracies were evaluated separately for overlapping accessions (*i*.*e*., 289 accessions planted in both years) and non-overlapping accessions (*i*.*e*., 234 newly added accessions in year 2).

### Expected improvement (EI)

In the previous study by Tanaka and Iwata (2018), EI of the *i*th accession was defined on the GBLUP model as follows:

5 $$EI_{i} = \left( {m_{i} - M} \right)\Phi \left( {z_{i} } \right) + s_{i} \phi \left( {z_{i} } \right)$$

6 $$z_{i} = \frac{{m_{i} - M}}{{s_{i} }}$$

where ${m}_{i}$ is the mean predicted genotypic value (i.e., PGV), ${s}_{i}$ is the standard deviation of the predicted genotypic value, and $M$ is the maximum estimated genotypic value among the observed genotypes. Further, $\phi$ and $\Phi$ are the probability density function and the cumulative density function of the standard normal distribution, respectively.

As its definition, this EI criterion becomes larger when PGV or its standard deviation (i.e*.*, prediction uncertainty) is larger, as higher standard deviation increases the chance of large genetic gain from the current best accession. Because EI is proportional to PGV given a prediction uncertainty, EI and PGV are highly related and top accessions in terms of EI or PGV may overlap.

There would be a few different ways to approximate or estimate the standard deviation of the predicted genotypic values. In this study, Eq. (4) used in the year 1 to year 2 selection was implemented using the BGLR package with 6000 iterations and 1200 burn-in period by sampling once per five iterations (Perez and de los Campos 2014). When using the Bayesian inference via the BGLR package, posterior standard deviation was returned in the summary object, calculated by using the MCMC samples after the burn-in period. Meanwhile, when Eq. (3) is used for the selection from year 2 to year 3, the mixed.solve function in the rrBLUP package was executed for solving the model via the restricted likelihood-based approach. By considering a conditional distribution of genotypic values given phenotypic values ignoring the fixed effects, standard deviations of the genotypic values were approximated as follows (Bishop 2006):

7 $$s = \left[ {\begin{array}{*{20}c} {s_{1} } & \cdots & {s_{3024} } \\ \end{array} } \right]^{T} \approx diag\left[ {\left( {Z^{T} Z\frac{1}{{\hat{\sigma }_{e}^{2} }} + G^{ - 1} \frac{1}{{\hat{\sigma }_{u}^{2} }}} \right)^{ - 1} } \right]$$

where ${\widehat{\sigma }}_{u}^{2}$ and ${\widehat{\sigma }}_{e}^{2}$ are the estimated genetic and residual variance, respectively, and the *diag* operator takes diagonal elements and makes a vector whose *i*th element is the (*i*, *i*)-element of the matrix inside the bracket. The above two methods are mathematically not equivalent (since the former is defined from a Bayesian perspective and the latter is from a frequentist perspective) but having a similar meaning, and therefore they will result in very close values. The former MCMC-based method is theoretically straightforward, as EI had been inspired by the Bayesian optimization algorithm. However, the latter was used when there were more than two fixed effects (i.e., when using year 1 + year 2 data) because calculation was faster when using rrBLUP.

### Phenotypic and genetic correlation analysis

As the objective of our study was to select accessions showing better performance across low-fertility fields in Madagascar, the GBLUP model was applied to predict genotypic values across sites. Thus, the genotype-by-site (GxS) interaction was not explicitly accounted for in the model (*i.e.*, it is included in the residuals). Nonetheless, it is of interest to evaluate the strength of GxS and genotype x year (GxY) observed in our experiment and for that purpose phenotypic and genetic correlations were calculated for every pair of year-site combination in year 1 and 2. Data from year 3 were excluded from this analysis because only 23 accessions overlapped between year 3 and other datasets.

Phenotypic correlations were calculated using Pearson’s correlation coefficients, by using the accessions whose phenotypic observations are available in both year-site combinations. For the genetic correlation, the GBLUP model was fitted in each year-site combination by using Eq. 4, to calculate estimated genotypic values of the 3 K accessions. For the subset of accessions tested in each year-site combination, we then calculated genetic correlations as Pearson’s correlations.

## Results

### Selection result for each trait

For the selection from year 1 to year 2, the GBLUP model (Eq. 4) was applied to each site (Anjiro and Behenjy) and for each trait (STW and TPW) independently, and PGV and EI were calculated for all untested accessions to select top 20 accessions for each criterion-site-trait combination. As there was a large overlap in the top accessions between the two criteria, in total 98 accessions was listed as final selection candidates. However, due to unavailability of seeds and other technical issues, the final number of newly selected and phenotyped accessions were 41 for TPW and 40 for STW. Similarly, the selection based on PGV and EI from year 2 to year 3 showed a large overlap, and in total 52 accessions were selected for TPW. We did not consider STW at that time. In this section, results are shown by merging the two criteria and the two sites of the training data. Overlap and difference among criteria and sites will be described later.

**Fig. 1:**
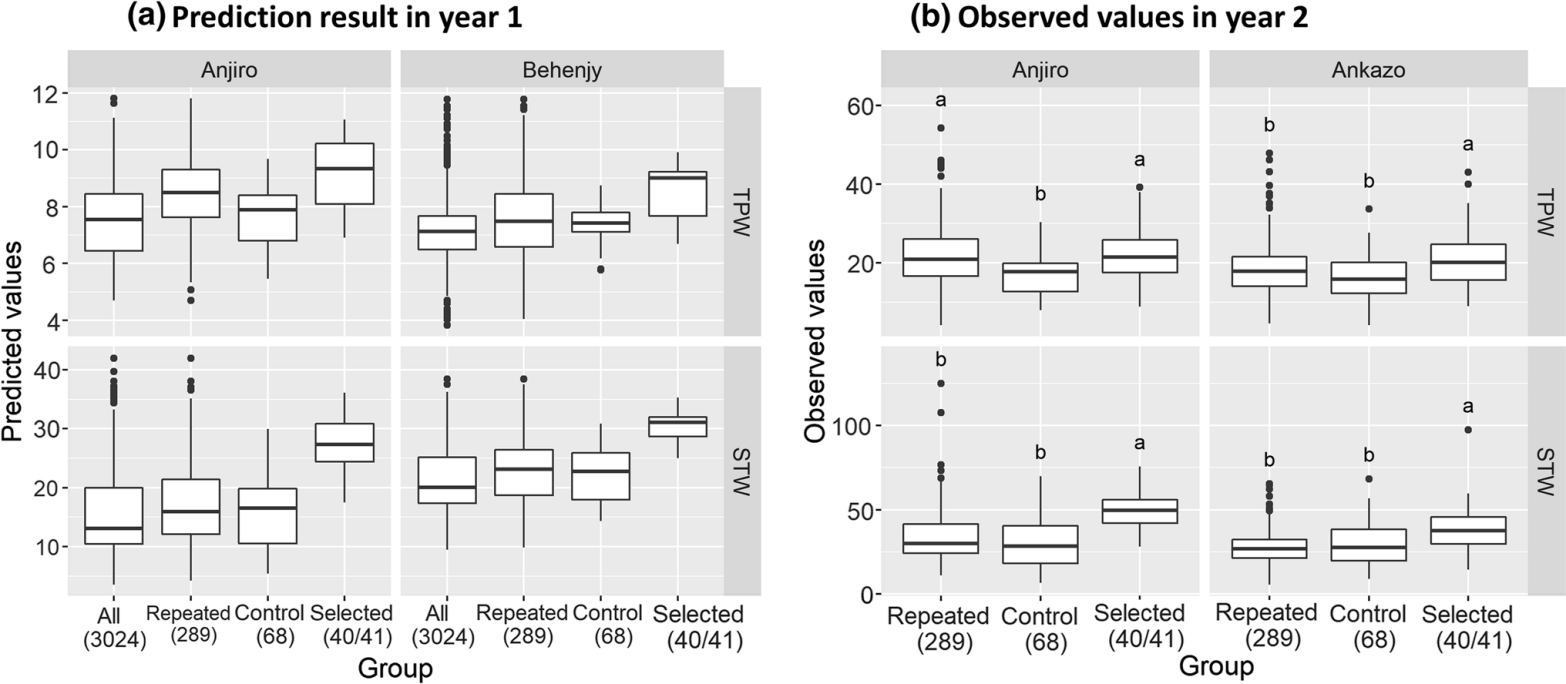
Selection result from year 1 to year 2. **a** Predicted genotypic values based on year 1 phenotypic values. **b** Observed phenotypic values in year 2. Selected accessions (*n* = 40 for total straw weight, STW; *n* = 41 for panicle dry weight, TPW) includes all accessions selected by predicted genotypic values (PGV) and expected improvement (EI), based on both Anjiro and Behenjy phenotypic values. For the observed values, Tukey HSD was applied for each combination of trait and site. Group with label “a” has significantly larger average than the group with label “b”

The selected group had a predicted average STW that was 78% (Anjiro) and 45% (Behenjy) higher compared to the average 3 K predicted values (Fig. 1a). For TPW, the predicted differences were smaller, about 21% and 19% higher in Anjiro and Behenjy, respectively. The control group (“Control” in Fig. 1a) medians were slightly higher compared to the 3 K average, but the overall distribution was similar (Fig. 1a; supplementary Figure S2). The group of accessions repeated in year 2 (“Repeated” in Fig. 1a) had similar predicted STW compared to the control and 3 K average, while their predicted TPW were slightly higher, particularly in Anjiro.

Figure 1b shows the actual performance in year 2 of the selected group *versus* the control and repeated groups. At both sites, TPW and STW were significantly higher in the selected (“Selected” in Fig. 1b) compared to the control group (“Control” in Fig. 1b) and this difference was more pronounced for STW. The repeated group of accessions (“Repeated” in Fig. 1b) performed similarly to the random control group for STW, while for TPW it contained a high number of well-performing accessions. In Anjiro, this led to significantly higher TPW compared to the control group (Fig. 1b).

**Fig. 2:**
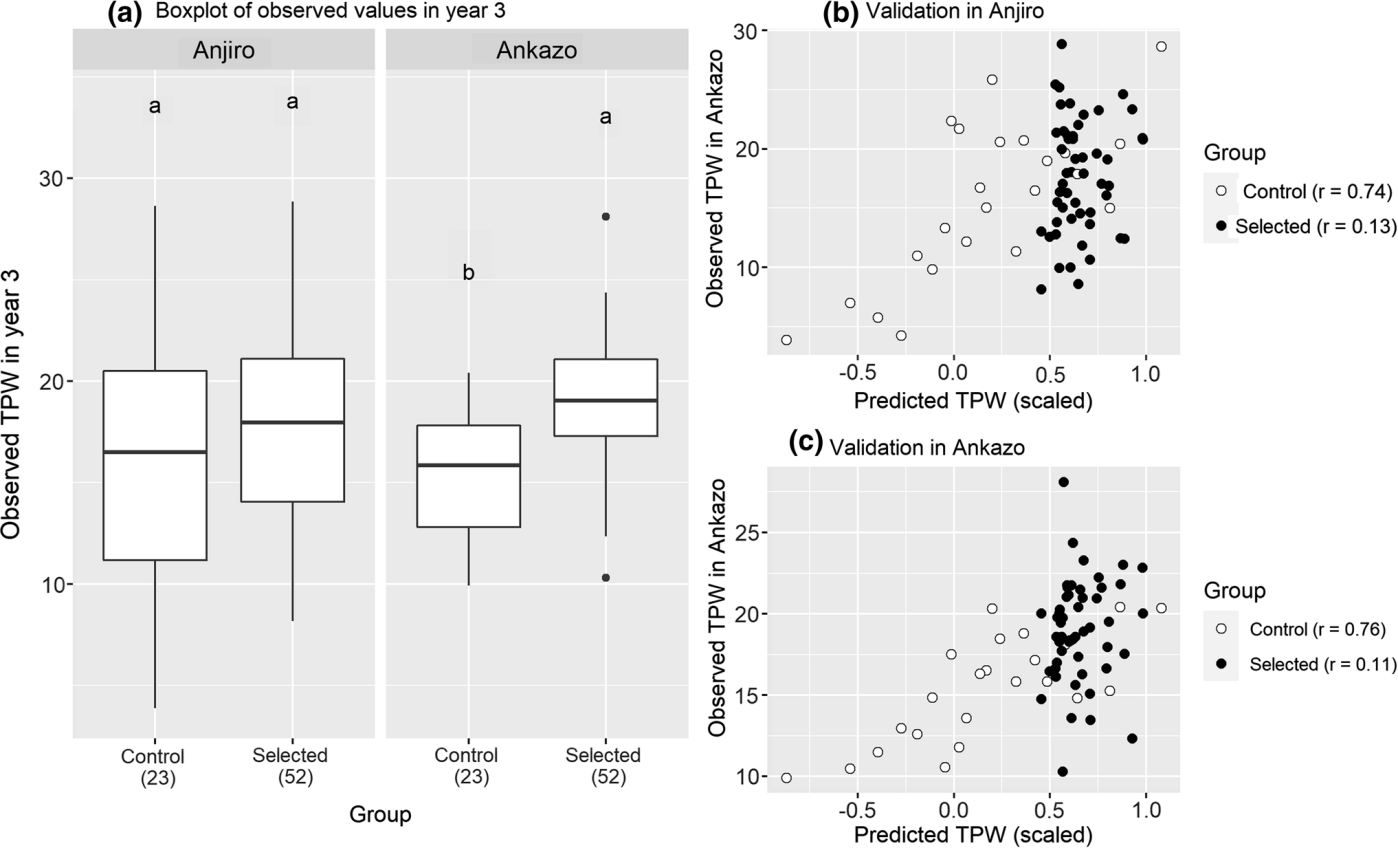
Selection result for total panicle dry weight (TPW) in year 3. Selection was based on TPW phenotype data in Anjiro year 1 and Ankazo year 2. **a** Boxplot of the observed TPW grouped by check (*n* = 23) and selected (*n* = 52) accessions. **b** Predicted and observed values in Anjiro. **c** Predicted and observed values in Ankazo

Selections from year 2 to year 3 were made for superior TPW using an updated prediction model that took year2 data from Ankazo (*n* = 523) into consideration in addition to year 1 data in Anjiro (*n* = 359) (supplementary Figure S1). The selected group (“Selected” in Fig. 2a) significantly outperformed the control group (“Control” in Fig. 2a) in Ankazo showing 22% higher observed TPW (Fig. 2a). In Anjiro, the average TPW was 14% higher, but due to large variation observed within selected and control groups this difference was not significant.

### Difference between selection criteria and sites to train the model

Results from prediction in year 1 validated in year 2, in which two sites (Anjiro and Behenjy) of training data were separately considered, were used to explain the difference among the selection criteria and sites. PGV and EI calculated from our model were positively correlated within a site and trait (supplementary Figure S3), and therefore the top accessions selected by these criteria largely overlapped. From year 1 to year 2, we listed the top 20 accessions for each trait and site predicted by the two criteria. If no overlap existed between the two criteria, 40 accessions would have been listed in each site for each trait. However, the overlap between criteria reduced accessions to 24 for STW in Anjiro (*i.e.*, 16 overlaps between PGV and EI) and to 28 in Behenjy (12 overlaps between PGV and EI), giving a total of 49 accessions across sites (3 overlaps between locations). Similarly, for TPW, 25 accessions were selected in Anjiro (15 overlaps between PGV and EI) while 26 accessions in Behenjy (14 overlaps), giving a total of 51 accessions (*i.e.*, no overlap between locations). There were 2 overlaps between the 49 and 51 accessions for STW and TPW.

**Fig. 3:**
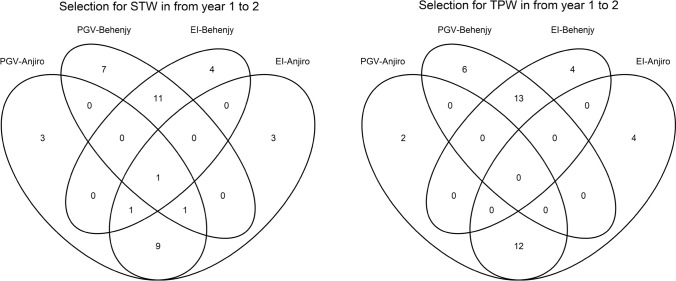
Overlap among the combination of selection methods and sites of the training data for total panicle dry weight (TPW) and PGV weight (STW), respectively. There was a large overlap between the two selection methods when applied to the same site

Because some of those 98 accessions were not available for distribution at IRRI, the final number of accessions tested was 41 for TPW and 40 for STW. Figure 3 summarizes the overlap of those 40 and 41 accessions. For example, there were 15 accessions newly evaluated according to the selection by PGV based on the Anjiro phenotype in year 1, while 9 accessions out of the 15 were also selected by EI based on the same phenotype data. Different accessions were selected when a different site was used as a training data. For the above example, only 1 common accession was selected based on Anjiro and Behenjy phenotype data, even though the same criterium (PGV) was used for the selection. Note that the total number of newly selected accessions based on PGV or EI in year 2 was 79, because there were 2 overlaps between the 41 and 40 accessions for TPW and STW.

**Fig. 4:**
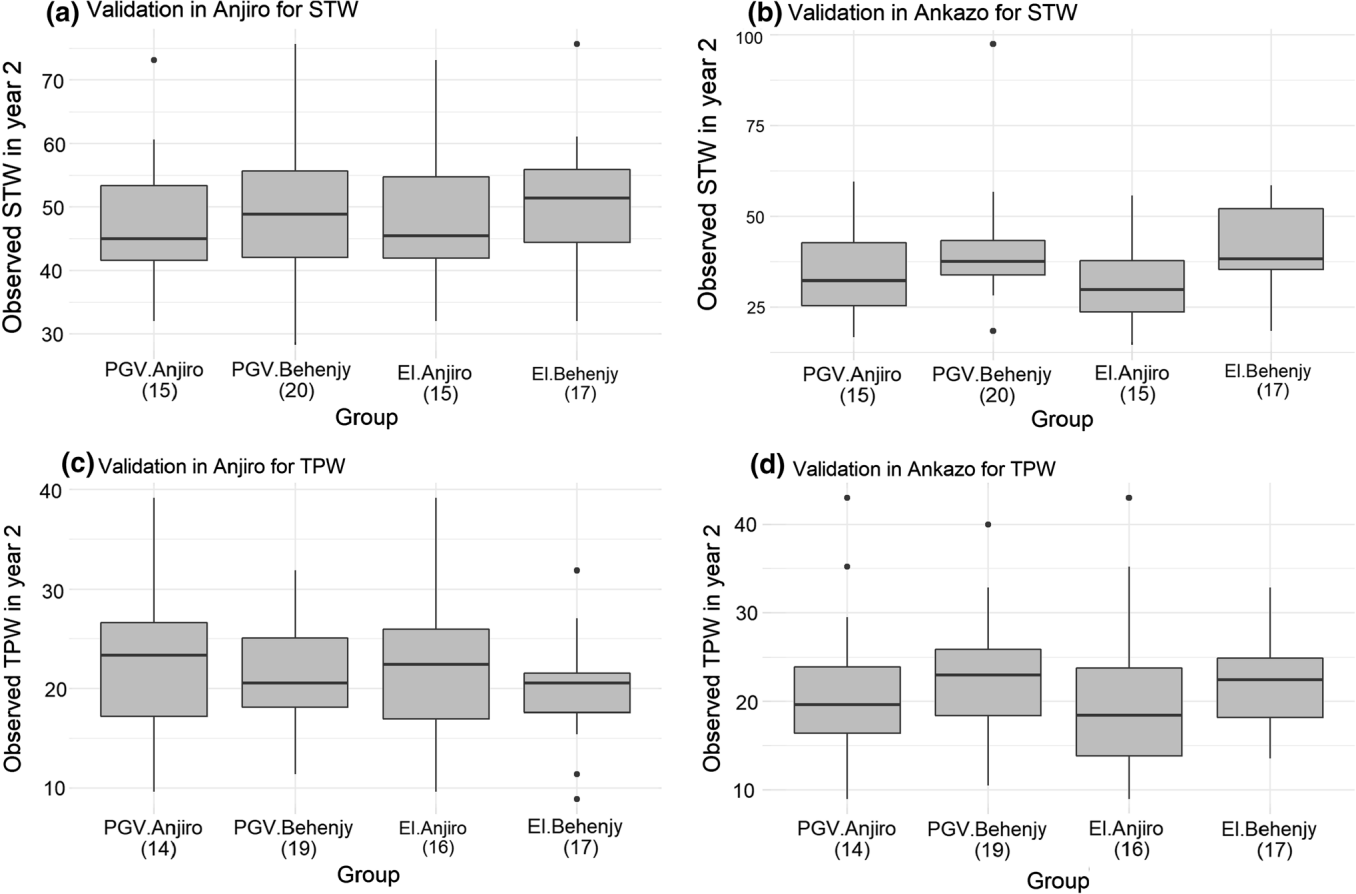
Observed phenotypic values grouped by selection criteria and site of the training phenotype data. There was no significant difference among the four groups in any figure

Figure 4 shows observed phenotypic values in year 2, grouped by selection criteria and sites of the training phenotypic data. Significant differences were not detected for any trait and testing environment (Tukey’s HSD at *P* < 0.05). However, there was a consistent trend suggesting selection based on Behenjy TPW and STW phenotype data resulted in better performance in Ankazo (Fig. 4b, d). For the second site (Anjiro), this trend was not consistent: Higher TPW was realized when the Anjiro phenotype was used for the selection, while higher STW was realized from selections based on Behenjy phenotype data (Fig. 4a, c).

The PGV and EI for year 2–3 selection for TPW were compared as well. As described in the Materials and Methods section, 42 and 45 accessions had been selected based on PGV and EI, respectively, with 35 accessions overlapping. Due to this large overlap, no difference between the two criteria was identified (supplementary Figure S4).

### Prediction accuracy

Prediction accuracy is an important factor for the success of genomic selection. Table 1 shows prediction accuracies estimated using tenfold cross-validation on year 1 or 2 phenotype data. Accuracy ranged from 0.29 for TPW in year 1 Anjiro to 0.75 for HD in year 2 Anjiro. The average accuracy for each trait across four year-by-site combinations was lowest for TPW (*r* = 0.38), intermediate for STW (*r* = 0.53), and highest for HD (*r* = 0.70). It is interesting that the accuracies for STW did not show a clear improvement from year 1 to 2, although the size of the dataset changed from *n* = 359 to *n* = 523 as new accessions had been added in year 2.

**Table 1:** Estimated accuracy using tenfold cross-validations. Numbers in brackets are standard deviations based on 10 replications

Trait	Year 1		Year 2	
	Anjiro	Behenjy	Anjiro	Ankazo
STW	0.61 (0.011)	0.47 (0.016)	0.57 (0.005)	0.50 (0.045)
TPW	0.29 (0.018)	0.43 (0.016)	0.37 (0.006)	0.45 (0.008)
HD	0.67 (0.011)	0.62 (0.008)	0.75 (0.005)	0.75 (0.003)

Although cross-validation is a common way to estimate the accuracy, it is more interesting and important to validate the prediction result from year 1 using the phenotypic values in year 2. Table 2 summarizes the prediction accuracies using year 1 phenotype as training data and year 2 phenotype as test data. The accuracies for accessions not included in the training dataset were as high or even higher than for repeatedly phenotyped accessions. For example, accuracy for STW in year 2 Anjiro based on the Anjiro phenotype in year 1 was 0.46 for accessions having been repeatedly phenotyped in year 1 and year 2 compared to 0.63 for newly included accessions. This is a surprising but promising result, because it indicates that predictions were accurate even for accessions lacking phenotypic data in a training dataset. The ranking of the prediction accuracy for different traits was identical (HD > STW > TPW) to the cross-validation result.

**Table 2:** Prediction accuracy of year 2 performance from year 1 training data in accessions that were either present in both years (repeated) or newly added (new) in year 2 based on their predicted superior performance

Trait	Test set	From Anjiro to Anjiro	From Anjiro to Ankazo	From Behenjy to Anjiro	From Behenjy to Ankazo
STW	Repeated (*n* = 289)	0.46	0.25	0.40	0.26
	New (*n* = 234)	0.63	0.46	0.63	0.49
TPW	Repeated (*n* = 289)	0.20	0.13	0.24	0.30
	new (*n* = 234)	0.22	0.10	0.24	0.24
HD	Repeated (*n* = 289)	0.73	0.65	0.73	0.72
	New (*n* = 234)	0.68	0.69	0.74	0.76

Best combination of training and testing site was highlighted by underbars

The prediction accuracy of TPW realized in year 3 based on year 1 and year 2 phenotype data (Eq. 3) was also tested even though the number of accessions evaluated in year 3 was small. It was found that the accuracy for the control group was high (*r* = 0.74 in Anjiro, *r* = 0.76 in Ankazo), while for the newly selected accessions it was low (*r* = 0.13 in Anjiro, *r* = 0.11 in Ankazo) (Fig. 2b, c). This may be because newly selected accessions were genetically less diverse compared to the control group, which was selected (from the training set) to contain a wide range of predicted performance. In fact, the accession with the highest predicted TPW was from the control set and it performed well in Anjiro but was surpassed by many newly selected accessions in Ankazo.

### Phenotypic and genetic correlation

Phenotypic correlations for TPW ranged from *r* = 0.10 (year 1 at Anjiro with year 2 at Ankazo) to *r* = 0.31 (year 1 at Behenjy with year 2 at Ankazo) and on average *r* = 0.21 (supplementary Table S 2). Correlation within sites (*r* = 0.23 between year 1 and 2 at Anjiro) were generally not higher than between sites (*e.g.*, *r* = 0.30 between Anjiro and Ankazo in year 2), possibly indicating that GxS effects were not stronger than GxY (genotype-by-year) effects. Similarly, phenotypic correlations for STW within sites between years and between sites within years appeared to be similar (e.g., *r* = 0.42 between years 1 and 2 at Anjiro compared to *r* = 0.42 between Anjiro and Behenjy at year1 or *r* = 0.43 between Anjiro and Ankazo in year 2). Phenotypic correlations for HD were moderate to high ranging from *r* = 0.64–0.92, confirming that HD is a highly heritable trait for which site and year effects are expected to be low.

As expected, genetic correlations were higher than phenotypic correlations. Genetic correlations ranged from *r* = 0.22–0.64 for TPW, from *r* = 0.44–0.69 for STW, and from *r* = 0.67–0.93 for HD (supplementary Table S 2). Interestingly, for all three traits, the lowest genetic correlation was observed between Anjiro year 1 and year 2 at Ankazo, and the highest genetic correlation was observed between Anjiro and Ankazo in year 2. This coheres with phenotypic correlations, suggesting that GxS may have been weak and that some GxY interaction affected outcomes.

## Discussion

Gene banks are considered a reservoir of untapped allelic variants that could improve the adaptation of crops to biotic and abiotic stresses (Brar and Khush 2018; McCouch et al. 2012). One of the biggest single crop gene banks is housed by IRRI, containing 132,000 accessions of cultivated *Oryza sativa, Oryza glaberrima*, and their wild relatives (https://www.irri.org/international-rice-genebank). Possibly the largest obstacle in exploiting these genetic resources in crop improvement is the lack of a systematic approach in evaluating this resource (McCouch et al. 2012). For most applications, it is simply not feasible to test all stored accessions, and sufficient information allowing for the selection of a representative subset typically does not exist. To overcome this limitation, IRRI and partners have selectively sequenced a cross section of 3024 of their *O. sativa* resources and made sequence data and seed publicly available (Mansueto et al. 2017).

Our general objective is to utilize this resource in breeding for adaptation to low soil fertility as one would encounter in small holder farmers’ fields in Madagascar and sub-Saharan Africa, where low soil fertility is compounded by insufficient fertilizer inputs (Tsujimoto et al. 2019; Saito et al. 2019). Most breeding programs are conducted on-station under high-fertility conditions, and this difference between target and selection environment is a potential obstacle in identifying new donors, loci, and alleles improving on-farm grain yield. Screening for new donors should therefore be conducted under target environment conditions; however, phenotyping all 3 K accessions in farmer’s fields in several locations is a formidable task that may not be an ideal use of resources, given the inherent variability in unfertilized farmer’s fields. Instead, one may test a subset of accessions and develop a genomic prediction model to predict the performance of the untested accessions, then only further test the best predicted accessions in the field. To what extent such an approach is feasible in selection for superior performance in off-station trials conducted in farmer’s fields was the specific objective of this study.

### Predicting and selecting superior gene bank accessions

The prediction accuracy of year 2 performance from year 1 training data differed considerably between traits with HD having the highest accuracy with an average of around 0.71 (Table 2) which was followed by the accuracy for shoot weight (ranging from 0.25 to 0.63) and TPW (0.10–0.30). This order matched the order for estimated broad-sense heritability, which was *H*^2^ = 0.95–0.98 for HD, *H*^2^ = 0.62–0.73 for STW and *H*^2^ = 0.56–0.61 for TPW (data not shown). Such a positive effect of increasing heritability on prediction accuracy is to be expected (Daetwyler et al. 2008) and has indeed been reported for rice in an empirical study (Spindel et al. 2015).

Compared to the difference in prediction accuracy between traits, the effect of sites was minor. Generally training data from the central Behenjy location was better able to predict TPW in Anjiro (East) and Ankazo (mid-West) compared to data from Anjiro, but for shoot weight and HD, differences between sites were small and inconsistent. The poor predictability of Ankazo TPW from year 1 Anjiro training data could have been due to high GxS or GxY effects. Although the low phenotypic and genetic correlations suggested the presence of GxS interactions for TPW, correlations between sites were in a similar range compared to correlations between years within sites. This implies that the performance of the accessions was under a shared genetic control among sites, particularly at Anjiro and Ankazo and that year effects may have been strong in same cases as for Anjiro year1, which was an untypically dry year during the early part of the season. Consistent GxS effects on the other hand appear to have been weak and this was expected considering all three sites in this study were P deficient low-input sites. Behenjy in year 1 was additionally limited by low temperatures, especially during the grain filling phase of late-maturing accessions. This known effect was purposely eliminated in year 2 by a) omitting very late maturing accessions as not adapted to Malagasy conditions, and b) selecting screening sites (Anjiro and Ankazo) for years 2 and 3 that do not experience this low temperature limitation.

Prediction accuracies were estimated for the set of accessions repeated in both years in comparison with the newly evaluated set (selected based on predictions). It may be expected that prediction accuracies would be higher for the repeated set as these accessions made up the training set; however, for HD and panicle weight no difference was observed, while the newly evaluated set had higher accuracies for shoot weight (Table 2). This may reflect a slightly narrower phenotypic variation within the repeated set compared to the newly evaluated set (supplementary Figure S5), which could have resulted from having omitted accessions having shown poor adaptation to local conditions in the first year.

Realized prediction accuracies for total PWT were in the range of 0.24–0.30 for training data from Behenjy and of 0.20–0.22 for Anjiro (in Anjiro). Comparable accuracies of *r* = 0.29 for panicle weight were reported from on-station trials conducted with a similar size training set at CIAT, Colombia, by Grenier et al. (2015). Similarly, on-station evaluations done at IRRI, Philippines, reported accuracies of up to *r* = 0.31 for grain yield (Spindel et al. 2015), whereas higher accuracies of *r* = 0.54 and 0.63 were detected for days to flowering in the wet and dry seasons, respectively. Conducting the phenotypic evaluations in farmers’ fields without fertilizer addition, mechanic land leveling, nor reliable irrigation therefore was able to provide a training dataset that could be used to predict field performance.

### Prediction criteria EI versus PGV

A simulation study showed that EI-based selection can discover superior accessions more efficiently than the usual PGV-based selection (Tanaka and Iwata 2018). We intended to test whether this proved correct in this study; however, no significant difference between EI and PGV in the ability to identify superior accessions was detected. One possible reason was the large training dataset (*n* = 359 in year 1, increasing to *n* = 523 in year 2) used here. Tanaka and Iwata (2018) argued that the difference between EI and PGV may decrease as the size of the training dataset increases, although their simulation did not show that trend. Since minimizing the training set size was not an objective of the current study, one likely outcome of employing a sizable training set was that prediction uncertainties were rather low, and as a result, EI and PGV selected a very similar set of accessions. Given our experimental/selection pipeline, it was difficult to make inference regarding the power of EI to provide an efficient genomic selection based on uncertain prediction result generated from smaller field phenotyping experiments.

A similar question related to maximizing selection gain given a limited budget is related to benefits of replicated phenotyping experiments. In year 1, field phenotyping was done without replication while year 2 experiments were replicated once at each site. Estimating the accuracy in both years through tenfold cross-validations indicated improvements of accuracies for TPW and HD in the year 2 dataset but these were minor, while the standard deviations of the accuracies were wider in year 1. Thus, predictions based on a non-replicated experiment in year 1 were not markedly inferior to those from the repeated year 2 experiments. This observation would match simulations of the benefits of replicated experiment versus increasing the size of the training set which concluded that adding replications may not be as effective as increasing training set size (Lorenz 2013).

### Optimization of the training set and the field experiment

In this study, we selected 359 initial accessions based on their origin, assuming a focus on accessions whose origins are in potentially similar environments to low-input medium elevation lowland paddy fields in Madagascar may increase chances of identifying superior performing one. To what extent this may have materialized is summarized in the form of group means by country of origin in supplementary Table S1. For TPW in Behenjy, the Madagascar group was superior followed by the group from Lao, whereas Indonesian and Sri Lankan accessions performed rather poorly. This is likely attributable to sensitivity to lower temperatures at the higher elevation site of the latter groups, which was also evident from the delayed heading of between 14.1 and 18.8 days in Behenjy relative to Anjiro. At the warmer Anjiro site, the country of origin did not have an effect on TPW. At both sites, the groups from Indonesia and Lao had highest straw weights, which could indicate that Indonesian accessions may have some adaptation to low soil fertility despite their low TPW.

A different approach in assembling the initial training set would be to aim for maximizing the prediction accuracy based on statistical methods such as PEV (prediction error variance) or CD (coefficient of determination) (Rincent et al. 2012; Akdemir et al. 2015; Akdemir and Isidro-Sanchez 2019). Further, recent studies empirically showed that accessions having higher *U*-value (upper bound of reliability) tend to show an extreme predicted value which can improve prediction accuracy (Yu et al. 2016, 2020) and contribute to selection of superior genotypes.

This optimization of the training set becomes a more complex but important problem when planning field experiments in multiple locations (i.e*.*, multi-environmental trial). We tested the same set of accessions in multiple locations. This is a straightforward experimental design which allows us to calculate phenotypic correlations among sites to evaluate the strength of GxS interaction. However, when genomic selection is used, it is no longer necessary to evaluate the same set of accessions across sites. Even though no accession is shared among environments, both GBLUP model and multi-environment genomic prediction models can be applied. Previous studies showed that multi-environment genomic prediction models can significantly improve the prediction accuracy from one environment to another (Burgueño et al. 2012; Cuevas et al. 2017; Guo et al. 2013; Lopez-Cruz et al. 2015; Mageto et al. 2020). This implies that it might be better to evaluate different sets of accessions in different environments, which is suggested as a “sparce test” in Jarquin et al. (2020).

In practice, accuracy in predicting the overall population (in our case, all 3 K accessions) is not necessarily the most important criterion for choosing an approach in genomic selection. For example, little is gained by a model predicting poorly performing accessions with high accuracy if this accuracy is not equally matched at the desired end of the distribution. The accuracy of predicting best performers has been assessed by a few studies (Ornella et al. 2014; Blondel et al. 2015), but the approach considered by these studies has not been commonly employed. It is therefore not resolved whether assembling a training set to maximize prediction accuracy is a better approach compared to assembling a training set based on anticipated adaptation due to geographical similarity.

While our study was not designed to answer above question, one may consider the evidence based on progress made in selecting superior performing accessions over the 2-year selection period. In predictions of the performance of 3 K accessions based on the model developed from year 1 data, a very high proportion of accessions from the year 1 training set were among the top accessions. Of the 60 top accessions (2% of the 3 K set) for STW in Anjiro, 29 had been part of the training set (8.1% of the training set). This increased to 42 accessions (11.7% of the training set) for predicted total panicle weight at Behenjy. This higher than expected proportion of top predicted accession in the training set may be caused by a shrinkage of the genomic prediction (i.e*.*, predicted values of the untested accessions tend to be biased toward the population mean), however, Fig. 1b indicates that many of the best-performing accessions in year 2 were from the repeat set. The performance of accessions in year 3 (Fig. 2b, c) further indicated that we were able to successfully predict and select superior accessions despite having a low prediction accuracy overall. It is interesting to note that two accessions have been chosen as donors in the Malagasy lowland rice breeding program. Donor IRIS 313–11949 (subpopulation: ind1A, origin: China) was part of the accessions newly selected in year 2. It showed 6th highest predicted TPW based on the Behenjy site data in year 1, and it was recommended by both EI and PGV. This accession subsequently proved to be among the top 3% performers for TPW across both sites in year 2, while having average STW and medium-early maturity. The second donor (IRIS 313–7832) was included in the initial set of 359 accessions (subpopulation: ind1B, origin: unknown) and was among the top 3 accessions at Ankazo but performed less well in Anjiro.

## Conclusions

How to accelerate the utilization of gene bank resources in crop improvement is an unresolved question and here we empirically tested whether a genomic prediction approach could aid in the selection of promising donors. Despite having conducted phenotypic evaluations under challenging conditions on smallholder farms, our results are encouraging as the prediction accuracy realized in these on-farm studies was in the range of accuracies achieved in on-station or even pure simulation studies. Thus, we could provide clear empirical evidence on the value of genomic selection in identifying suitable genetic resources for crop improvement, if genotypic data are available. The approach taken by IRRI and partners of making such data publicly available for at least 3,024 of their gene bank accessions represents an important step toward more efficient utilization of these gene bank resources.

### Supplementary Information

Below is the link to the electronic supplementary material.

Flowchart describing the different sets of materials used in evaluations and predictions over the 3-year experimental period (PPTX 97 KB)

Predicted values of newly added accessions in year2, in addition to the EI/PGV-based selection. Sets of 68 control accessions (blue dots) and 87 accessions (red dots) were selected to enhance the training data of genomic prediction. The 68 controls showed a similar distribution to the entire 3K accession (PPTX 222 KB)

Relationship between predicted genotypic values (PGV) and expected improvement (EI) for the four trait-environment combinations. Both were calculated for the untested (i.e., not phenotyped in year 1) subset of the 3K accession (PPTX 173 KB)

Observed panicle dry weight (TPW) of the selected accessions in 2018, grouped by the selection method. There was no significant difference between the two methods. Note that the two groups were largely overlapped (PPTX 89 KB)

Distribution of the observed phenotypic values in year 2. Accessions were grouped by repeatedly measured and newly evaluated ones (PPTX 109 KB)

Supplementary file6 (DOCX 15 KB)

Supplementary file7 (XLSX 12 KB)

## Data Availability

Genotypic data used in the study are publicly available at https://snp-seek.irri.org/_snp.zul.
