# Correction: From gene banks to farmer’s fields: using genomic selection to identify donors for a breeding program in rice to close the yield gap on smallholder farms

**DOI:** 10.1007/s00122-024-04622-z

**Published:** 2024-05-10

**Authors:** Ryokei Tanaka, James Lui-King, Sarah Tojo Mandaharisoa, Mbolatantely Rakotondramanana, Harisoa Nicole Ranaivo, Juan Pariasca-Tanaka, Hiromi Kajiya Kanegae, Hiroyoshi Iwata, Matthias Wissuwa

**Affiliations:** 1 Department of Agricultural and Environmental Biology, Graduate School of Agricultural and Life Sciences, The University of Tokyo, 1-1-1 Yayoi, Bunkyo, Tokyo 113-8657 Japan; 2 International Program in Agricultural Development Studies, Graduate School of Agricultural and Life Sciences, The University of Tokyo, 1-1-1 Yayoi, Bunkyo, Tokyo 113-8657 Japan; 3 Crop, Livestock and Environment Division, Japan International Research Center for Agricultural Sciences (JIRCAS), 1-1 Ohwashi, Tsukuba, Ibaraki 305-8686 Japan; 4 Rice Research Department, The National Center for Applied Research On Rural Development (FOFIFA), Antananarivo, 101 Madagascar

**Correction: Theoretical and Applied Genetics (2021) 134:3397–3410** 10.1007/s00122-021-03909-9

The original version of this article omitted to include information about a co-author. The co-author James Lui-King should have been listed among the authors of the manuscript and in the author contribution statement. Also, the Monsanto Beachell-Borlaug International Scholars Program (MBBISP) should have been mentioned under Funding information. The original article has been corrected accordingly. The corrected statements are given below.

