# Supplementary material for: From gene banks to farmer’s fields: using genomic selection to identify donors for a breeding program in rice to close the yield gap on smallholder farms
Source: Theor Appl Genet. 2021 Jul 15;134(10):3397–410. doi: 10.1007/s00122-021-03909-9 (PMC8440315; doi:10.1007/s00122-021-03909-9)
Supplement: Supplementary file 1 — Flowchart describing the different sets of materials used in evaluations and predictions over the 3-year experimental period (PPTX 97 KB) [file 122_2021_3909_MOESM1_ESM.pptx]

## Slide 1
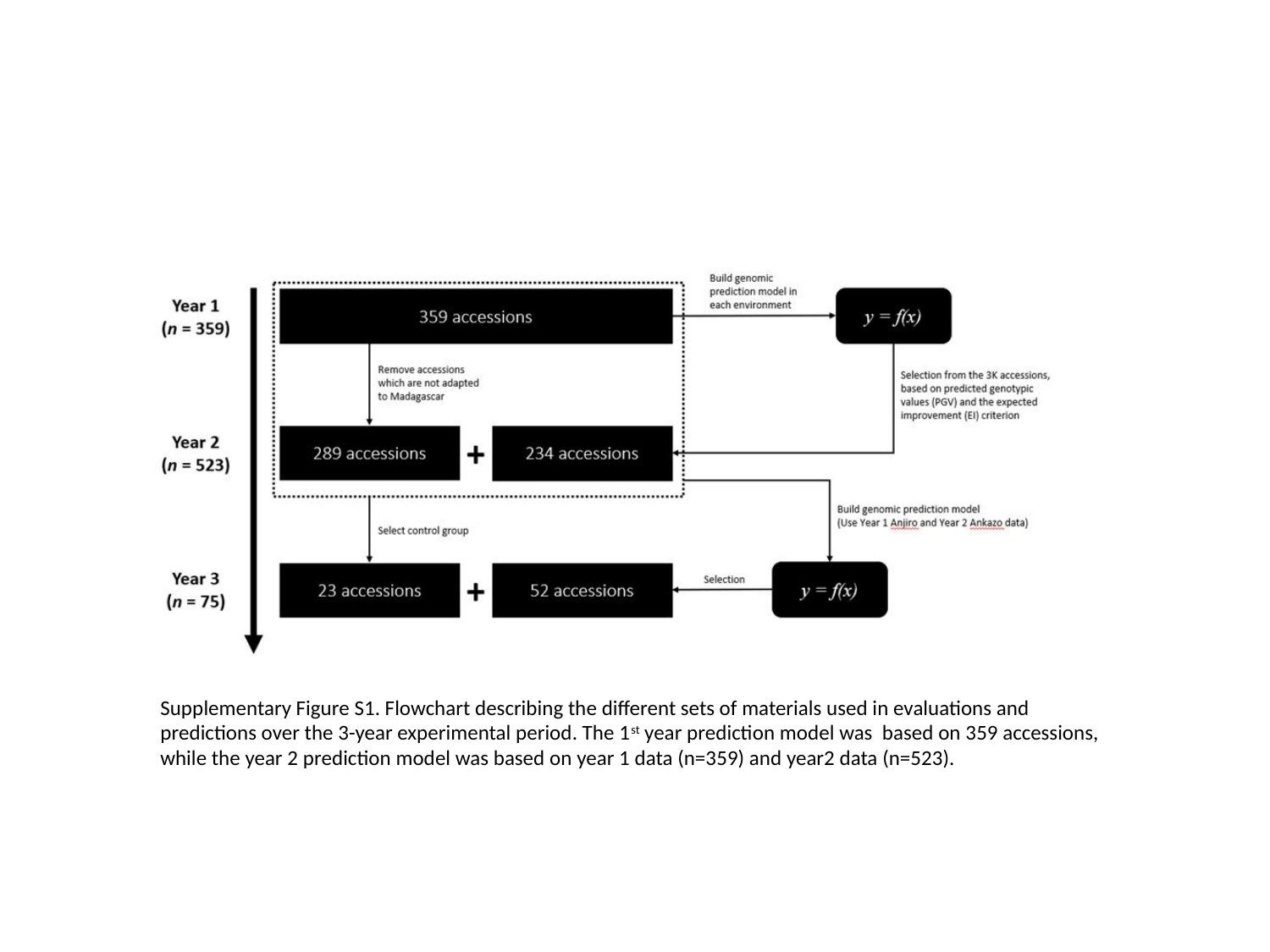

Supplementary Figure S1. Flowchart describing the different sets of materials used in evaluations and predictions over the 3-year experimental period. The 1st year prediction model was based on 359 accessions, while the year 2 prediction model was based on year 1 data (n=359) and year2 data (n=523).
