# Supplementary figures and images for: From gene banks to farmer’s fields: using genomic selection to identify donors for a breeding program in rice to close the yield gap on smallholder farms
Source: Theor Appl Genet. 2021 Jul 15;134(10):3397–410. doi: 10.1007/s00122-021-03909-9 (PMC8440315; doi:10.1007/s00122-021-03909-9)

## Slide 1
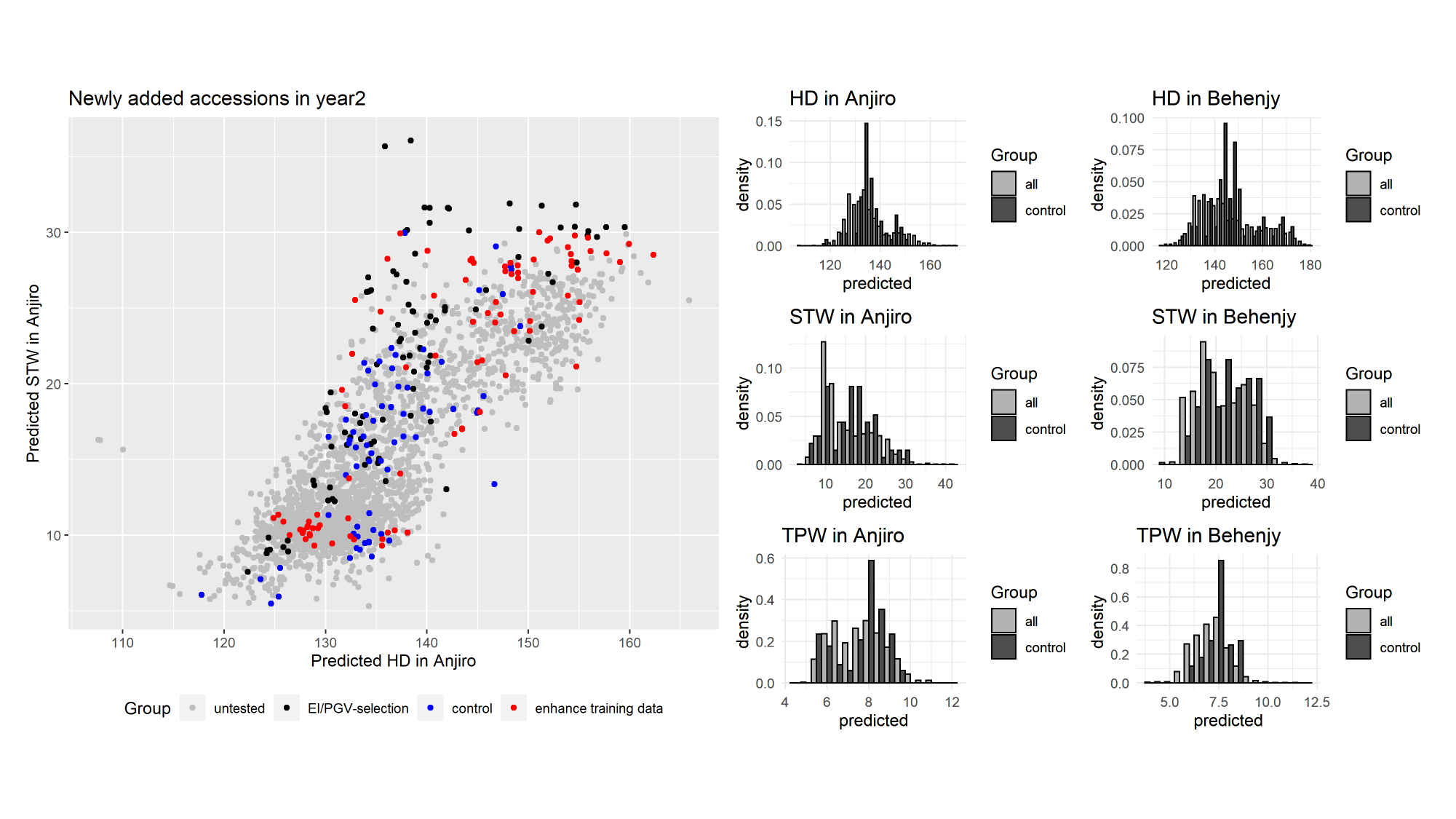

Supplement: Supplementary file 2 — Predicted values of newly added accessions in year2, in addition to the EI/PGV-based selection. Sets of 68 control accessions (blue dots) and 87 accessions (red dots) were selected to enhance the training data of genomic prediction. The 68 controls showed a similar distribution to the entire 3K accession (PPTX 222 KB) [file 122_2021_3909_MOESM2_ESM.pptx]

## Slide 1
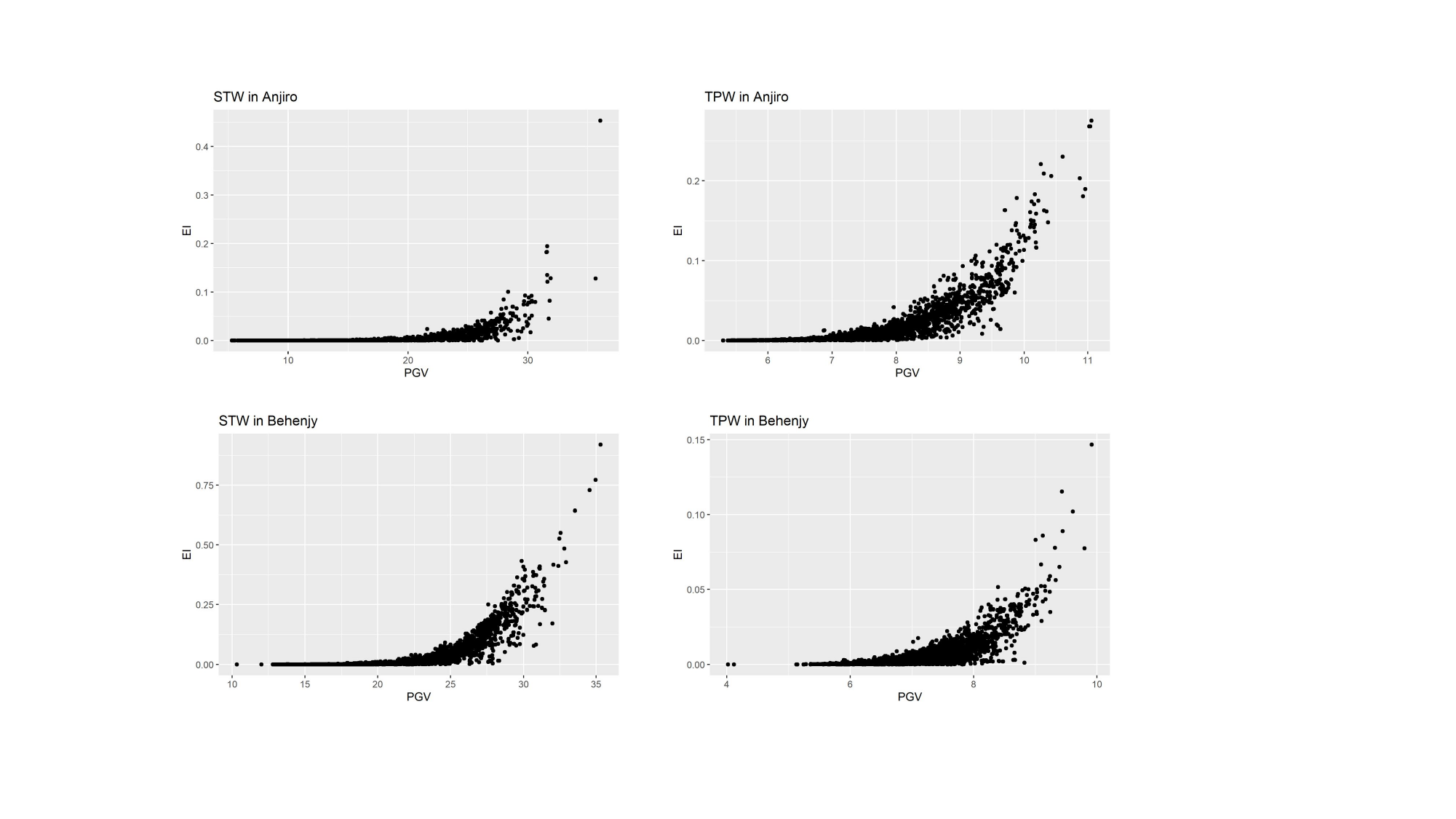

Supplement: Supplementary file 3 — Relationship between predicted genotypic values (PGV) and expected improvement (EI) for the four trait-environment combinations. Both were calculated for the untested (i.e., not phenotyped in year 1) subset of the 3K accession (PPTX 173 KB) [file 122_2021_3909_MOESM3_ESM.pptx]

## Slide 1
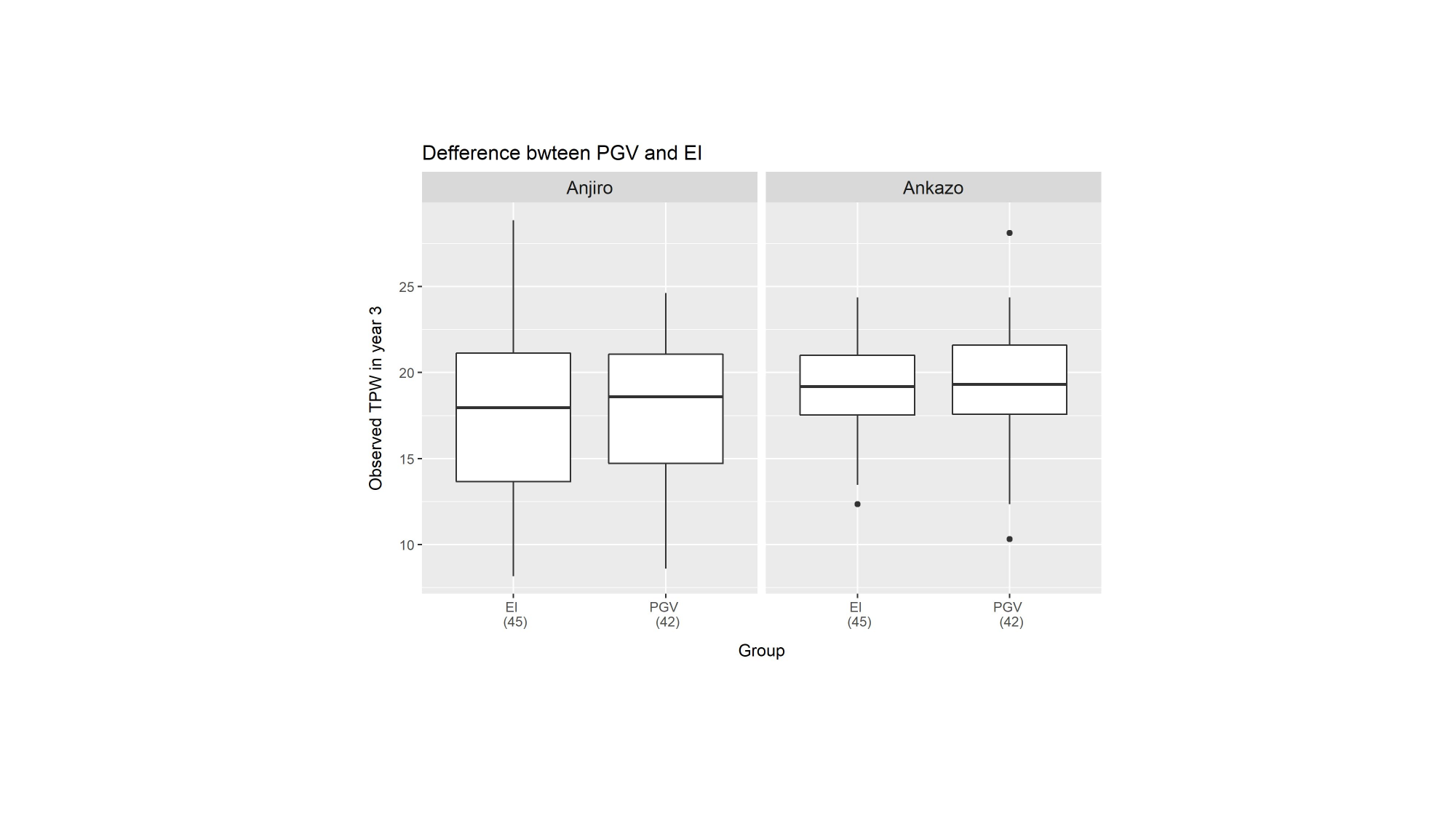

Supplement: Supplementary file 4 — Observed panicle dry weight (TPW) of the selected accessions in 2018, grouped by the selection method. There was no significant difference between the two methods. Note that the two groups were largely overlapped (PPTX 89 KB) [file 122_2021_3909_MOESM4_ESM.pptx]

## Slide 1
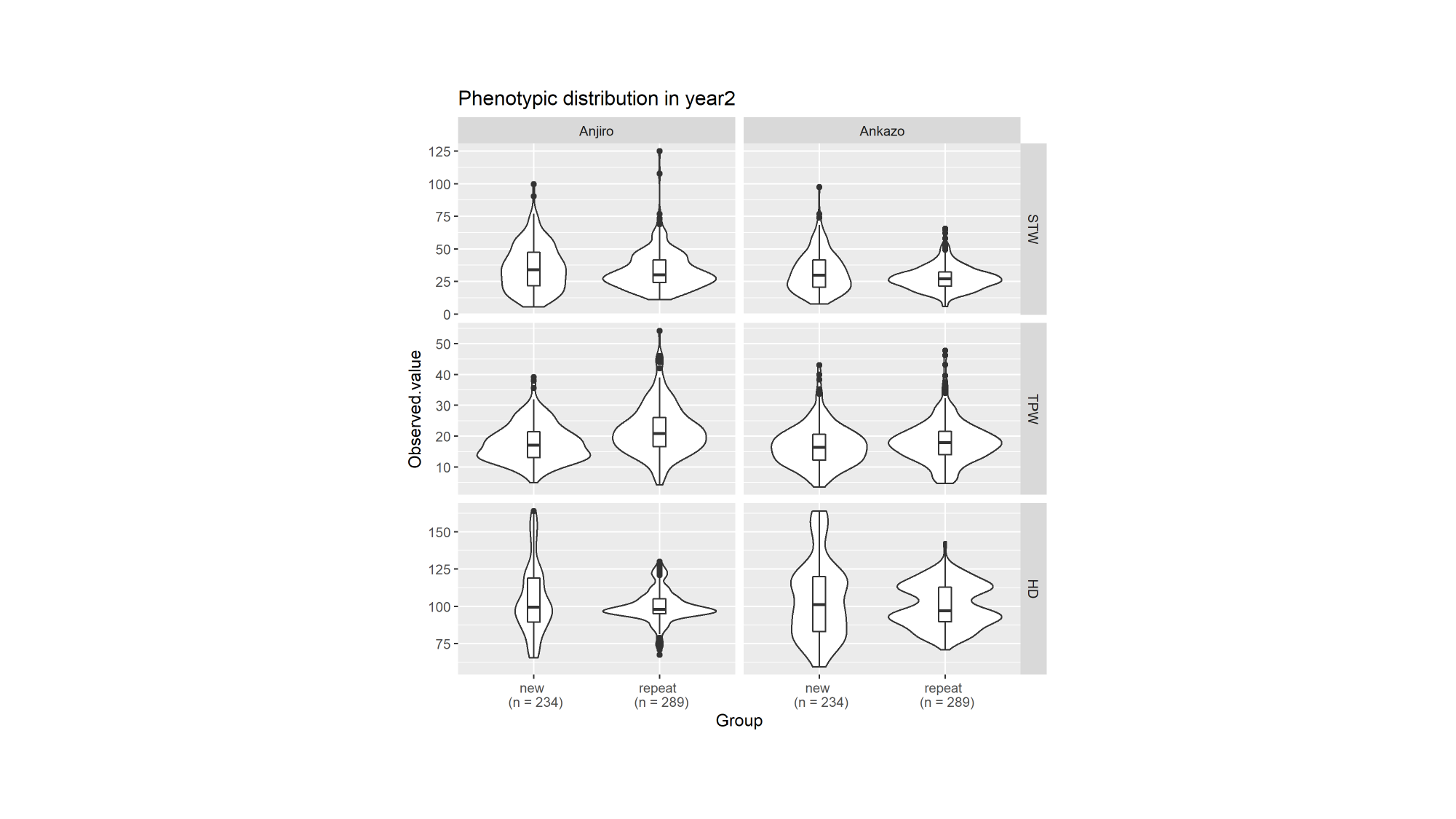

Supplement: Supplementary file 5 — Distribution of the observed phenotypic values in year 2. Accessions were grouped by repeatedly measured and newly evaluated ones (PPTX 109 KB) [file 122_2021_3909_MOESM5_ESM.pptx]
