# Supplementary material for: From gene banks to farmer’s fields: using genomic selection to identify donors for a breeding program in rice to close the yield gap on smallholder farms
Source: Theor Appl Genet. 2021 Jul 15;134(10):3397–410. doi: 10.1007/s00122-021-03909-9 (PMC8440315; doi:10.1007/s00122-021-03909-9)
Supplement: Supplementary file 6 — Supplementary file6 (DOCX 15 KB) [file 122_2021_3909_MOESM6_ESM.docx]

Supplementary Table S1. Average total panicle weight (TPW, in g plant^-1^), straw weight (STW, in g plant^-1^) and heading date (HD, in days) in accessions grouped by country of origin (year 1 data). The non-local group contains all accessions from outside of Madagascar while the Nepali group also contains accessions from Bhutan.

|  |  |  | Behenji |  |  | Anjiro |  |
| --- | --- | --- | --- | --- | --- | --- | --- |
| Origin | n | TPW | STW | HD | TPW | STW | HD |
| Madagascar | 58 | 9.3 | 24.9 | 146.9 | 8 | 17.7 | 136.3 |
| Indonesia | 39 | 5.1 | 26.5 | 164 | 8 | 26.5 | 149.9 |
| Sri Lanka | 43 | 6.5 | 23.2 | 158.3 | 8 | 18.7 | 139.5 |
| Nepal | 55 | 7.2 | 19.3 | 134.3 | 7.3 | 12.1 | 127.6 |
| Lao | 31 | 8 | 27.9 | 143.2 | 8.9 | 20.8 | 133.7 |
| IRRI | 38 | 6.8 | 17.9 | 143.6 | 7.8 | 11.3 | 133.5 |
| non-local | 301 | 7.2 | 21.8 | 149.8 | 8.3 | 17.8 | 146.9 |
| HSD (0.05) |  | 1.6 | 6 | 9.3 | 1.7 | 4.6 | 5.5 |
